# Characterization of Papillary Thyroid Microcarcinomas Using Sonographic Features in Malignant Papillary Thyroid Cancer: A Retrospective Analysis

**DOI:** 10.1097/MD.0000000000000841

**Published:** 2015-05-29

**Authors:** Wei-jun Gu, Hui-xian Yan, Yu-kun Luo, Fu-lin Wang, Guo-qing Yang, Qing-hua Guo, Nian Jin, Li Zang, Kang Chen, Jin Du, Xian-ling Wang, Li-juan Yang, Jian-ming Ba, Jing-tao Dou, Yi-ming Mu, Chang-yu Pan, Zhao-hui Lv

**Affiliations:** From the Department of Endocrinology (WG, HY, GY, QG, NJ, LZ, KC, JD, XW, LY, JB, JD, YM, CP, ZL), PLA General Hospital; Beijing Haidian Hospital (HY); Department of Ultrasonography (YL); and Department of Pathology (FW), PLA General Hospital, Beijing, China.

## Abstract

The diagnosis of malignant thyroid nodules is still a clinical challenge. This study aimed to determine the ultrasonographic characteristics of papillary thyroid carcinoma.

The ultrasonographic and pathological data of 2453 thyroid nodules in a cohort of 1895 Chinese patients who underwent thyroidectomy from January 2010 to December 2012 were retrospectively reviewed.

Anteroposterior and transversal (AP/TR) diameters ≥1, solid structure, infiltrative margins, hypoechoic appearance, and microcalcifications were more common in malignant nodules than in benign nodules (*P* < 0.01). These ultrasonographic features were independent risk factors of malignancy (*P* < 0.01) as determined by logistic regression analysis. Based on multivariate analysis, these characteristics were also present in large nodules (diameter >10 mm). However, in small nodules (diameter ≤10 mm), only AP/TR ≥1 and infiltrative margins were independent risk factors of malignancy (*P* < 0.01).

Ultrasonography is of high diagnostic value for malignant thyroid nodules and may help to improve the differential diagnosis. Small and large nodules have distinct ultrasonographic features.

## INTRODUCTION

Papillary thyroid carcinoma (PTC) is the most common form of thyroid cancer, accounting for 75% to 85% of all cases of thyroid cancer.^1^ PTC is characterized with large or small thyroid nodules. Papillary thyroid microcarcinoma (PTMC) is a subset of PTC and is characterized with a maximum diameter measuring ≤10 mm.^1^ Thyroid nodules are highly common in the general population, and the majority of thyroid nodules are detected incidentally during physical examination or radiographic imaging.^2,3^ The estimated prevalence of thyroid nodules is as high as 20% to 70% in the general population, and thyroid nodules have a 5% to 15% prevalence of malignancy.^2,4,5^

At present, the malignancy of nodules is usually determined by fine-needle aspiration cytology (FNAC) or surgical biopsy. The use of FNAC has resulted in an increased frequency of PTMC diagnoses. However, this technique is invasive and associated with some complications. Ultrasound (US) is recommended as the preferred tool in the evaluation of thyroid nodules, because of its cost-effectiveness, availability, limited discomfort to the patient, and nonionizing nature. Thyroid US has been used to guide FNAC and differentiate benign and malignant nodules.^6^

Although several US features are associated with an increased risk of malignancy, the sensitivity varies in different studies.^2,3,5^ Recently, it has been reported that small and large thyroid nodules have different odds ratio (OR) of ultrasonic features for predicting malignancy.^6–8^ There has been no large-scale analysis of ultrasonographic features of thyroid nodules in the Chinese population. The aims of this retrospective study were to determine the ultrasonographic characteristics of malignant thyroid nodules and investigate the difference in PTC-associated US features between large (diameter >10 mm) and small nodules (diameter ≤10 mm).

## MATERIALS AND METHODS

### Patients

The Ethical Committee of the PLA General Hospital, Beijing, China, approved this retrospective study. This study was based on 1895 consecutive patients who underwent thyroidectomy from January 2010 to December 2012 at the Department of General Surgery, PLA General Hospital. The cohort included patients with both preoperative ultrasound and postoperative biopsy results. A total of 2453 thyroid nodules were retrospectively reviewed. Postoperative biopsy confirmed 1726 benign and 727 malignant nodules. Patients with non-PTC malignant nodules (follicular carcinoma or medullary carcinoma; n = 39) were excluded from the study. Therefore, 688 histopathologically diagnosed PTC nodules were included in this study.

### Patient Evaluation

All patients underwent a thyroid nodule US examination within 1 month before surgery. FNAC was performed only for patients with suspicious US features. Surgical indications were thyroid nodule(s) confirmed by ultrasound with suspicious US features: microcalcifications, infiltrative margins, anteroposterior/transversal (AP/TR) diameters ≥1, solitary, and hypoechoic; large goiter with findings suggestive of malignancy or indeterminate/suspicious FNAC finding; and no contraindications for surgical procedure.

A nodule was diagnosed as malignantly suspicious when at least 1 of the above ultrasonographic features was present. If >1 nodule was found under US, the same sonographer individually evaluated each nodule in 1 session. Nodules were histopathologically diagnosed as benign or papillary carcinoma, and then were further divided into 2 groups according to size: large nodules (diameter >10 mm) and small nodules (diameter ≤10 mm).

### US Examination Technique

All nodules were evaluated using a color Doppler sonography Philip iE33 (Philips Medical Systems, Bothell, WA) with 6 to 8 MHz or 10 to 12 MHz transducers. The 10 to 12 MHz transducer was applied for thin and superficial thyroid nodules and the 6 to 8 MHz transducer for posteriorly located nodules. Examinations were conducted and recorded by a skilled sonographer in accordance with a standard procedure. Nodules were described according to the following parameters: the maximum diameter; solid, mixed, or cystic echostructure; none, hypo-, iso-, hyper-, or mixed echogenicity; no calcification, microcalcification, coarse calcification, isolated edge, isolated, or no description; well-defined or infiltrative margins; AP/TR <1 or AP/TR ≥1; and no, less, or rich blood flow within the nodule or peripheral tissue.

### Statistical Analysis

Data are expressed as means ± standard deviation. Statistical analyses were performed with SPSS version 18.0 (SPSS Inc., Chicago, IL). *P* value of <0.05 was considered significant. Student *t* test was used to examine the differences between ultrasonographic parameters. Frequency distributions were compared with the χ^2^ squared test. Logistic regression analysis was used to assess the predictive ability of each US parameter for PTC. Univariate analysis was used to assess the relationship between sonographic parameters and histologic diagnosis; statistically significant variables were further analyzed by multiple logistic regression analysis. In addition, using the forward method of multiple logistic regression analysis, the interrelationship among the parameters was analyzed to determine the most predictive factors for PTC. Sensitivity, specificity, positive predictive value (PPV), negative predictive value, and accuracy were calculated to evaluate the diagnostic performance of each US feature.

## RESULTS

### General Characteristics of Benign and PTC Nodules

Based on histopathology, there were 1726 benign (70.4%) nodules and 727 malignant nodules. The malignant nodules were subdivided into 688 (28.0%) PTC nodules and 39 (1.6%) non-PTC follicular-type papillary carcinoma nodules (Table 1). The non-PTC nodules consisted of 19 medullary carcinoma nodules, 12 follicular carcinoma nodules, 5 anaplastic carcinoma nodules, 2 malignant lymphoma nodules, and 1 metastatic carcinoma.

**TABLE 1:**
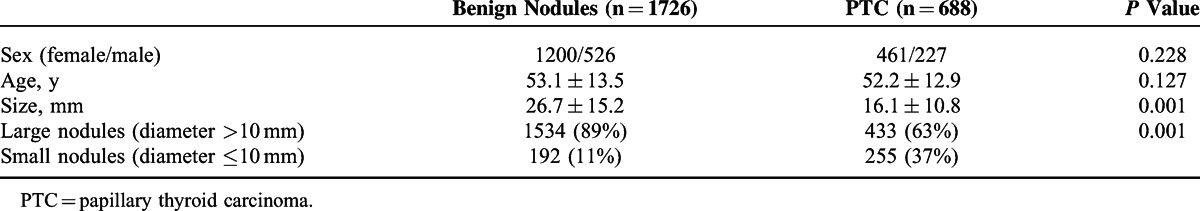
General Characteristics of Benign and PTC Nodules

Among the 2414 nodules (benign and PTC nodules), 753 (31.2%) were from male patients and 1661 (68.8%) were from female patients. The ratio of nodules from male-to-female subjects is 1:2.2. The mean age of all patients was 52.9 years (range 6–92 years). The mean age of male patients was 53.1 years (range 13–81 years), and the mean age of female patients was 52.2 years (range 6–92 years).

There were 1967 (81.5%) large nodules and 447 (18.5%) small nodules. The overall mean size of the nodules was 23.9 mm (range 2–74 mm). The mean size of PTC nodules was smaller than that of the benign nodules (16.1 vs 26.7 mm, *P* < 0.001). Within benign nodules, 1534 (88.8%) were large and 192 (11.1%) were small. Within PTC nodules, 433 (62.9%) were large and 255 (37.1%) were small (Table 1). In the large nodules, the mean diameter of PTC nodules was significantly smaller than that of benign ones (21.1 mm [range 11–84 mm] vs 29.2 mm [range 11–98 mm], *P* < 0.001). In the small nodules, the mean diameter of PTMC nodules was significantly larger than that of benign ones (7 mm [range 3–10 mm] vs 7.6 mm [range 3–10 mm], *P* < 0.001).

### US Characteristics

We observed a total of 885 (36.1%) single nodules and 1568 (63.9%) multiple nodules. In the benign nodules, the single and multiple nodule proportion was 33.2% and 66.8%, respectively. In the PCT nodules, the single and multiple nodule proportion was 42.9% and 57.1%, respectively, showing a higher proportion of the single nodules (Table 2). Within all the nodules (benign and PTC), 133 (5.4%) had cystic, 747 (30.5%) had solid, 725 (29.6%) had mixed structure, and 848 (34.5%) had no description of echostructure. Based on echogenicity of all the nodules, we observed 1062 (43.3%) hypoechoic, 136 (5.5%) isoechoic, 144 (5.9%) hyperechoic, 75 (3.1%) echoless, 781 (31.8%) cases with mixed echo, and 255 (10.4%) had no description. Of all nodules, 1344 (54.3%) had well-defined margins, 513 (20.9%) had infiltrative margins, and 596 (24.3%) had no description. Microcalcification was present in 418 nodules and nodular AP/TR was ≥1 in 456 patients.

**TABLE 2:**
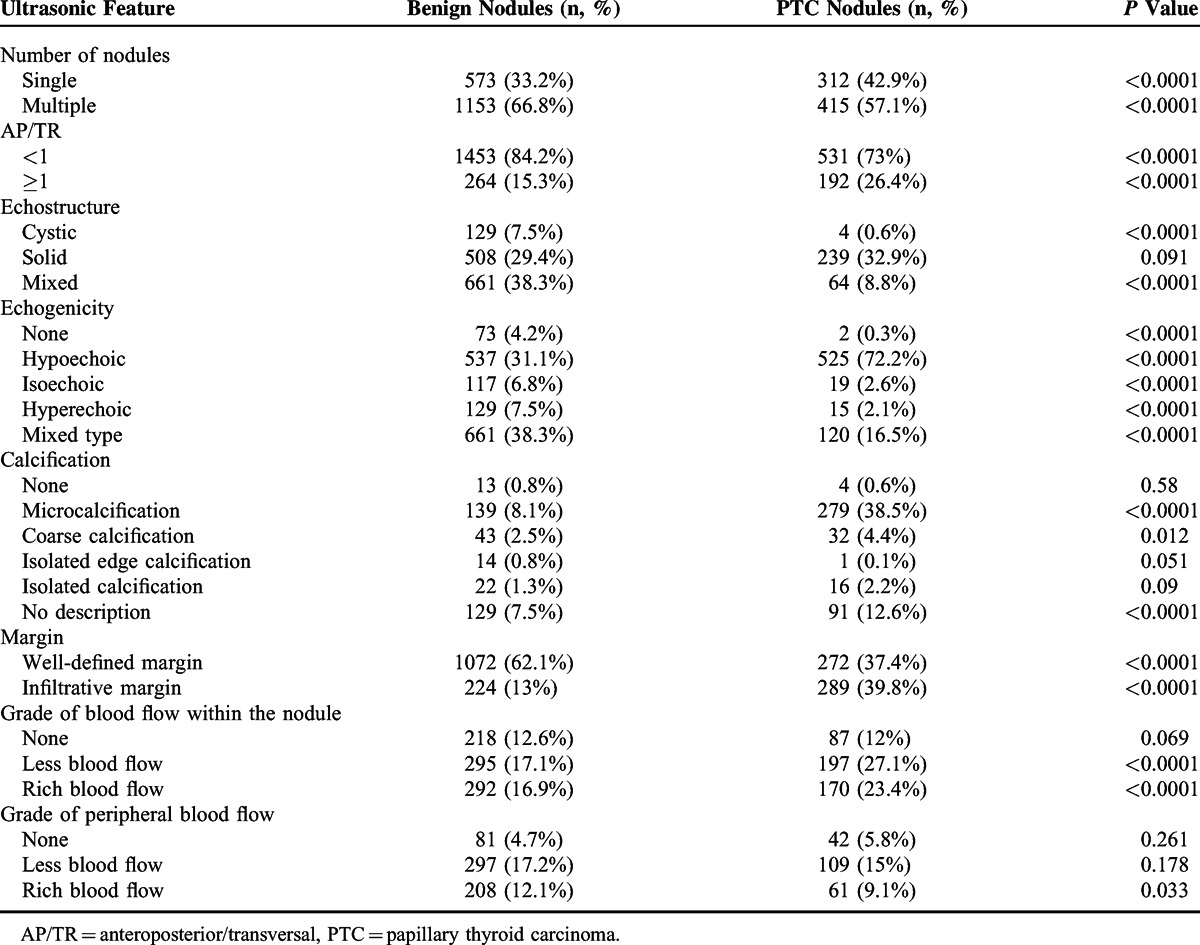
Ultrasonographic Parameters of Benign and PTC Nodules

### Difference in Sonographic Features Between Benign and PTC Nodules

The incidences of the following sonographic characteristics were significantly higher (*P* < 0.01) in the PTC nodules than in the benign nodules: singular nodule (42.8% vs 33.4%), AP/TR ≥1 (25.9% vs 15.4%), hypoechoic appearance (72.2% vs 31.1%), microcalcification (39.1% vs 8.1%), infiltrative margins (40.6% vs 13%), no vascularity (26.9% vs 17.1%), and rich vascularity (22.8% vs 16.9%) (Table 2). No statistical significance was detected in solid nodules between the 2 groups (Table 2).

### Univariate and Logistic Regression Analysis of US Features in PTC Nodules

Univariate analysis showed that the following US features were associated with significantly lower risk of PTC: multiple nodules, cystic or mixed nodules, and rich peripheral vascularity (Table 3). The following US features were associated with increased risk of PTC: AP/TR ≥1, solid structure, hypoechoic appearance, microcalcifications, infiltrative margins, and decreased vascularity or rich vascularity within the nodule (Table 3).

**TABLE 3:**
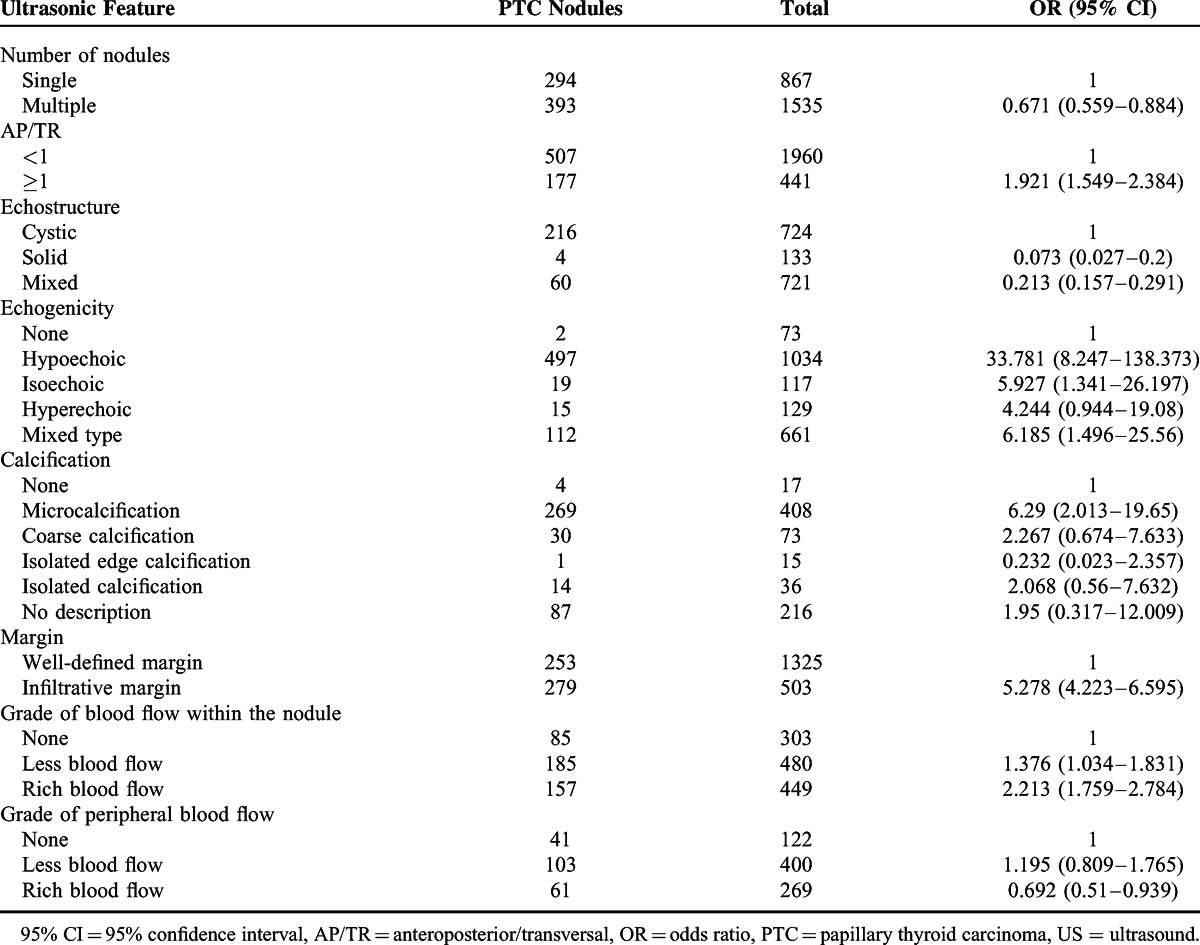
Univariate Analysis of US Features in PTC

Logistic regression analysis showed that the following features were independent determinants of PTC (*P* < 0.05): AP/TR ≥1, solid composition, infiltrative margins, hypoechoic appearance, and microcalcifications (Table 4). Of these features, hypoechoic appearance (OR = 6.548, *P* = 0.025) and microcalcifications (OR = 7.126, *P* = 0.002) were the most significant predictive factors.

**TABLE 4:**
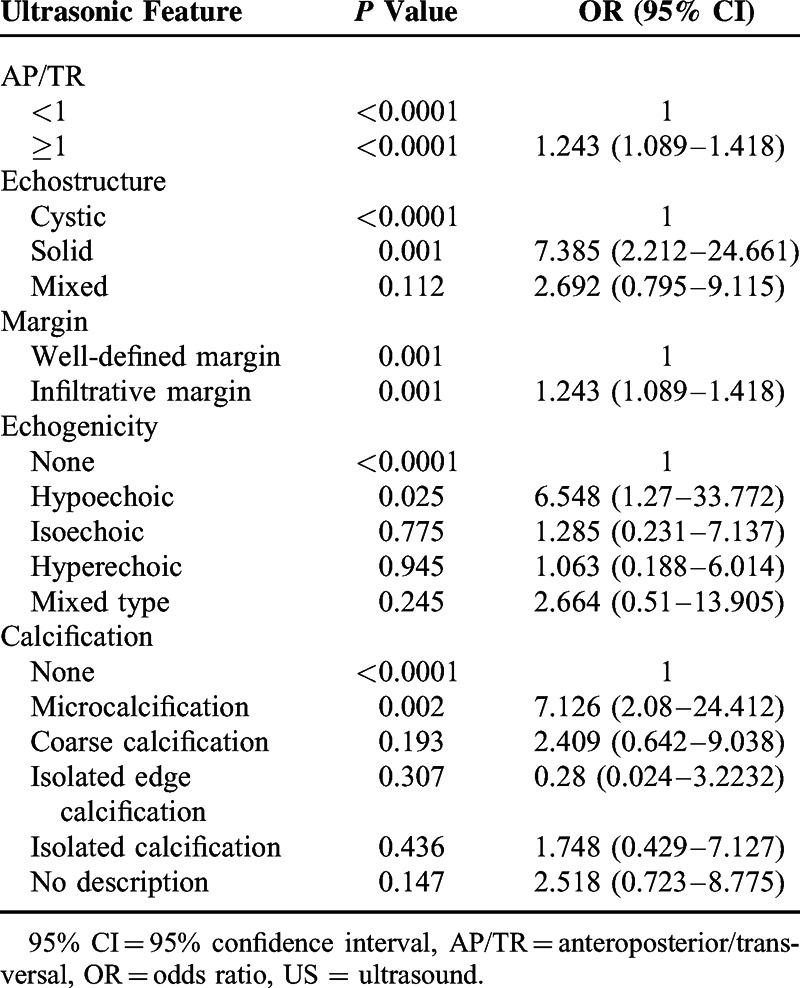
Multiple Regression Analysis of US Features in PTC

### Univariate and Logistic Regression Analysis According to Nodule Size

Logistic regression analysis was performed to analyze the differences between large and small nodules. Univariate analysis showed that all features (AP/TR ≥1, solid structure, infiltrative margins, hypoechoic appearance, and microcalcifications) were independent determinants of PTC in large nodules (*P* < 0.05). However, all but microcalcifications were independent determinants of PTC in small nodules (*P* < 0.05).

Multivariate analysis showed that in the large nodules, AP/TR ≥1 (OR = 1.532, *P* < 0.05), solid structure (OR = 3.111, *P* < 0.001), infiltrative margins (OR = 3.395, *P* < 0.001), hypoechoic appearance (OR = 5.403, *P* < 0.001), and microcalcifications (OR = 9.979, *P* < 0.001) were independent determinants of PTC. However, in the small nodules, only AP/TR ≥1 (OR = 1.779, *P* < 0.05) and infiltrative margins (OR = 3.936, *P* < 0.01) were independent risk factors of PTC. The sensitivity of AP/TR ≥1, infiltrative margins, solid composition, microcalcifications, and hypoechoic appearance in diagnosing PTC nodules were 63.0%, 51.5%, 88.0%, 65.5%, and 77.1%, respectively, and their specificity was 84.0%, 82.7%, 60.9%, 62.0%, and 64.6%, respectively.

### Analysis of US Features According to Nodule Size

Univariate and multivariate analysis showed that the following US features differed significantly between the PTC and benign nodules: AP/TR ≥1, solid composition, infiltrative margins, hypoechoic appearance, and microcalcifications. These 6 US features were regarded as suspicious features of PTC nodules (Table 4). The numbers of PTC nodules were significantly higher than benign nodules in both small and large nodules when ≥3 suspicious US features were used (*P* < 0.001) (Table 5). Furthermore, a significant difference between the large PTC nodules and PTMC nodules was detected when there were ≤3 suspicious US features (*P* < 0.001). The prevalence of malignant nodules was higher in small nodules than in large ones (*P* < 0.001). There was no significant difference between the PTC of small and large nodules when there were >4 suspicious US features (*P* > 0.05) (Table 5).

**TABLE 5:**
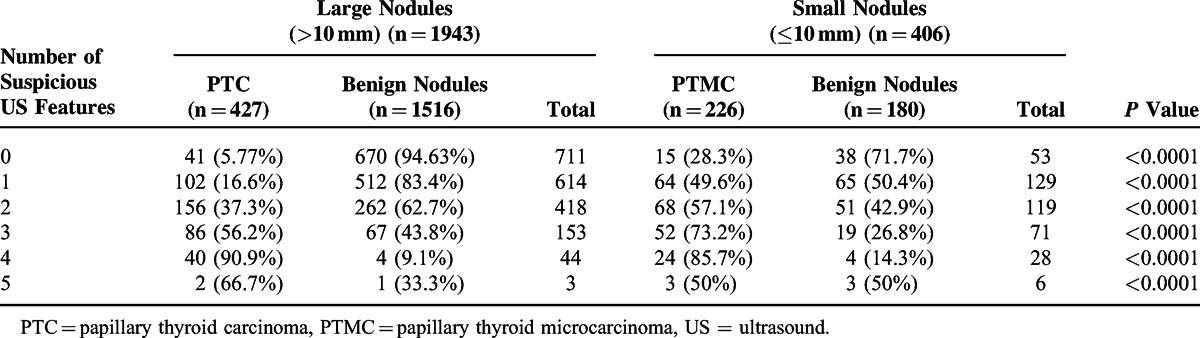
Number of Suspicious US Features According to Nodule Size

## DISCUSSION

Although most thyroid nodules are benign, clinicians must distinguish between malignant and benign nodules. Malignant nodules require timely surgical treatment, whereas most patients with benign thyroid nodules can be managed conservatively. Therefore, accurate diagnosis of thyroid nodules is vital for treatment and prognostic assessment. In our study, of all the 2414 consecutive thyroid nodules, only 688 (28.5%) were confirmed to be malignant by histopathology. Therefore, “overtreatment” should be avoided, as the high prevalence of thyroid nodules will significantly increase the physical and economic burden in a large number of patients.

Surgery is the main treatment for thyroid cancer. However, surgery is not only invasive but also causes a series of complications, such as recurrent laryngeal nerve injury and permanent hypoparathyroidism, which seriously affects survival and quality of life. Therefore, accurate preoperative assessment of malignancy is necessary to avoid excessive treatment in benign nodules and misdiagnoses in malignant nodules. At present, thyroid nodules are evaluated using many methods, such as ultrasonography, FNAC, emission computed tomography, computed tomography, and magnetic resonance imaging. Although cytology is an effective preoperative diagnostic method, its clinical application is limited by invasiveness. Ultrasonography is highly sensitive, cost-effective, convenient, and noninvasive, and has been used as the preferred screening method for suspect thyroid nodules.^9^

Preoperative US features can provide useful information for prediction of histological type and guide the proper management of thyroid nodules. Many studies have shown that several US features, such as the number of nodules, nodular margins, blood flow distribution, hypoechoic appearance, and microcalcification are significantly associated with PTC malignancy, regardless of nodule size.^10–12^ In our study, infiltrative margins, AP/TR ≥1, and solid composition were independent predictive factors of PTC (*P* < 0.01), but increased blood flow distribution (central or peripheral) was not. Other authors have also reported less predictive value of intranodular blood flow distribution.^13^ Berker et al^14^ and Lyshchik et al^15^ reported hypoechoic appearance as the only reliable malignant sign, but they did not evaluate the relationship between nodular margins and malignant, and all the patients in their studies were under the age of 18.

Leenhardt et al^16^ showed that hypoechoic appearance had moderate predictive value, a sensitivity of 75%, and a specificity of 61% to 75%. In our study, hypoechogenicity had a sensitivity of 77.1% and a specificity of 64.6%, similar to the findings of other study.^17^ Difference in AP/TR between benign and malignant nodules may be associated with the growth characteristic of nodules. Malignant nodules are invasive and the attenuation of the rear results in greater change in the diameter; microinfiltration surrounding malignant nodules also increases AP/TR. Calcification of thyroid nodules is a common ultrasound finding, and different calcification patterns have different clinical significance. Microcalcification can be highly indicative of thyroid carcinoma,^18,19^ and peripheral calcification, also known as “egg shell” calcification, commonly seen in benign nodules, could also be found in PTC. Our study found that 57.6% of thyroid cancer nodules had calcification, whereas calcification was present in only 20.1% of benign nodules. Malignant nodules were more likely to have calcification than benign nodules, and microcalcification was associated with thyroid carcinoma. Our study showed that microcalcification has a sensitivity of 65.5% and a specificity of 62% in diagnosing thyroid carcinoma.

Several studies have evaluated US features according to nodule size. Cappelli et al^20^ observed that the relationship between US features and malignancy were similar in large and small nodules. Kim et al^21^ found that an irregular margin was the best predictive criterion for malignancy in both large and small nodules, with an especially high OR in large nodules. Hypoechoic appearance and AP/TR ≥1 were independent risk factors for malignancy in small nodules.^21^ We also found differences in US features according to nodule size. Multivariate analysis of small nodules showed that only infiltrative margins and AP/TR ≥1 were independent determinants of PTC, whereas microcalcifications, hypoechogenicity, and solid composition were not. Similar to our results, Moon et al^22^ reported that fewer malignant nodules ≤10 mm had microcalcification than larger nodules, and that the diagnostic value of microcalcification was greater for large nodules than for small nodules.^22^ Differentiation of microcalcifications from colloid crystals might be difficult in some nodules <10 mm, and this may be one of the reasons for the lower accuracy of sonographic observation in the small nodules.

It has been shown that solid or half-solid nodules are associated with higher risks of malignancy than cystic nodules, but the PPV is relatively low, with only 15% to 27% in solid hypoechoic nodules.^17^ In our study, 32.9% of solid nodules were malignant, and the sensitivity and specificity of solid nodules were slightly higher than other compositions. We also found that the number of suspicious US features was significantly higher in PTC than in benign nodules, regardless of nodule size (*P* < 0.001). These results were consistent with the previously reported.^20^

Our study has some limitations. There was unavoidable selection bias because of the lack of histological data in ultrasonically benign patients. FNAC was not routinely performed in diagnostic work-up before surgical procedure as recommended by many guidelines. The retrospective study design prevented real-time evaluation of US findings, and interpretation may vary among different sonographers. Not all nodules in our study were histologically confirmed by surgical biopsy.

In conclusion, the OR of each US finding for predicting PTC is different between large and small nodules. Not all sonographic features can be applied to microcarcinomas for risk prediction of PTC. Therefore, it would be helpful to consider the importance of certain US features according to nodule size when assessing risk of PTC in thyroid nodules.
